# Phylogenomic analyses of multidrug resistant *Corynebacterium striatum* strains isolated from patients in a tertiary care hospital in the UK

**DOI:** 10.1007/s10096-024-04857-0

**Published:** 2024-05-27

**Authors:** Vartul Sangal, Emma C. L. Marrs, Andrew Nelson, John D. Perry

**Affiliations:** 1 Faculty of Health and Life Sciences, Northumbria University, Newcastle upon Tyne, UK; 2 Microbiology Research Department, Freeman Hospital, Newcastle upon Tyne, UK

**Keywords:** Antimicrobial resistance, *Corynebacterium striatum*, Nosocomial infection, Patients, Phylogenomic diversity, Virulence

## Abstract

*Corynebacterium striatum* is an emerging nosocomial pathogen. This is the first report showing the presence of three distinct multidrug resistant lineages of *C. striatum* among patients in a UK hospital. The presence of ErmX, Tet(W), Bla and AmpC proteins, and mutations in *gyrA* gene are associated with the resistance to clindamycin, doxycycline, penicillin and moxifloxacin, respectively. These strains are equipped with several corynebacterial virulence genes including two SpaDEF-type and a novel pilus gene cluster, which needs further molecular characterisation. This study highlights a need of developing an active surveillance strategy for routine monitoring and preventing potential cross-transmission among susceptible patients.

**Supplementary Information:**

The online version contains supplementary material available at 10.1007/s10096-024-04857-0.

*Corynebacterium striatum* is a common inhabitant of human skin and mucosal membranes but is emerging as an important opportunistic pathogen, especially for people with underlying health conditions both in the community and healthcare settings [1–3]. Multidrug resistance (MDR) among these strains has further exacerbated the situation resulting in high mortality among patients [3–5]. However, clinical surveillance and epidemiological monitoring of this pathogen in healthcare settings are not well established, and molecular mechanisms of virulence and MDR phenotypes are poorly characterised. This is the first study showing the presence of three distinct MDR *C. striatum* lineages in a UK healthcare setting.

Nine *C. striatum* strains were isolated from patients admitted to the Newcastle upon Tyne Hospitals NHS Trust between October 2021 and May 2022 with a range of infections including bone infection, corneal ulcer, osteomyelitis, suspected urinary tract infection and septic arthritis (Table 1). Two strains were isolated from patients with infected pacemaker and bilateral ureteric stents. Eight of the patients were adults (average age: 71 years) and one was an infant. Six of the nine patients had underlying health conditions, which is consistent with the opportunistic nature of this pathogen.

**Table 1:** List of clinical *C. striatum* isolates

Strain ID	Year of isolation	Patient details		Source	Clinical diagnosis	Underlying conditions
		Sex	Age range (Y)			
708C	2021	M	60–70	Joint fluid	Septic arthritis	Lung cancer
600M	2021	M	70–80	Bone	Infected bone	Diabetes
640X	2021	M	50–60	Corneal scrape	Corneal ulcer	None
719S	2021	F	80–90	Pacing wires	Infected pacemaker	Coronary artery disease
821F	2022	M	70–80	Tissue	Osteomyelitis	Diabetes
821A	2022	F	80–90	Sphenoid swab	Osteomyelitis	None
824M	2022	F	0–5	Blood	Fever	Tumour / neutropenia
391E	2022	M	70–80	Kidney urine	Bilateral ureteric stents	None
397Q	2022	F	50–60	Catch urine	Suspected UTI	Neutropenia

Genomic DNA were extracted from 1.5 ml overnight brain-heart infusion broth (Oxoid, UK) cultures using the DNeasy PowerSoil Pro Kit (Qiagen, UK) and were sequenced on a MiSeq instrument (Illumina, USA). 300 bp paired-end reads were assembled using Spades 3.13.1 [6] and scaffolded using Multi-CSAR web-server [7] with five complete genomes (Accession: NZ_CP024931.1, NZ_CP024932.1, NZ_CP068158.1, NZ_CP068157.1 and NZ_CP069514.1) as references. We obtained 345 publicly available *C. striatum* genome sequences from the GenBank (Accessed on 14/07/2023) but excluded one genome with < 90% completeness and ≥ 10% contamination [8]. All genomes were annotated using Prokka 1.13.7 [9] and were compared using Roary 3.12.0 [10]. A maximum-likelihood tree from core genomic alignment was constructed using IQ-tree 1.6.11 [11], that was re-rooted on the longest branch using iTOL server [12]. The clades with average distance from nodes to leaves below 0.015 were assigned group designations and the remaining strains were treated as singletons.

Phylogenomic analysis revealed 14 groups and 13 singleton strains (Fig. 1). Four UK strains, 708C, 600M, 821A and 391E formed a subgroup (UK-A) within Group-2 with strains from Canada, China, Germany and USA (Fig. 1). These strains were isolated from diverse sources including blood, body fluids (bronchial alveolar lavage, joint fluid, endotracheal and tracheal aspirates), swabs (tissue, wound, bone, foot ulcer and sphenoid swab), urine and sputum samples (Table S1). Three UK isolates 824M, 719S and 821F (UK-B) clustered in Group-4 with strains from Australia and USA, again mostly from diverse invasive sources including a pacing wire from a patient (Table 1, Table S1). The remaining two isolates, 640X and 397Q (UK-C) grouped with two isolates from China and one from Denmark in Group-14 (Fig. 1). These results indicate the existence of three distinct lineages of *C. striatum* strains in the tertiary care hospital in the UK.

**Fig. 1:**
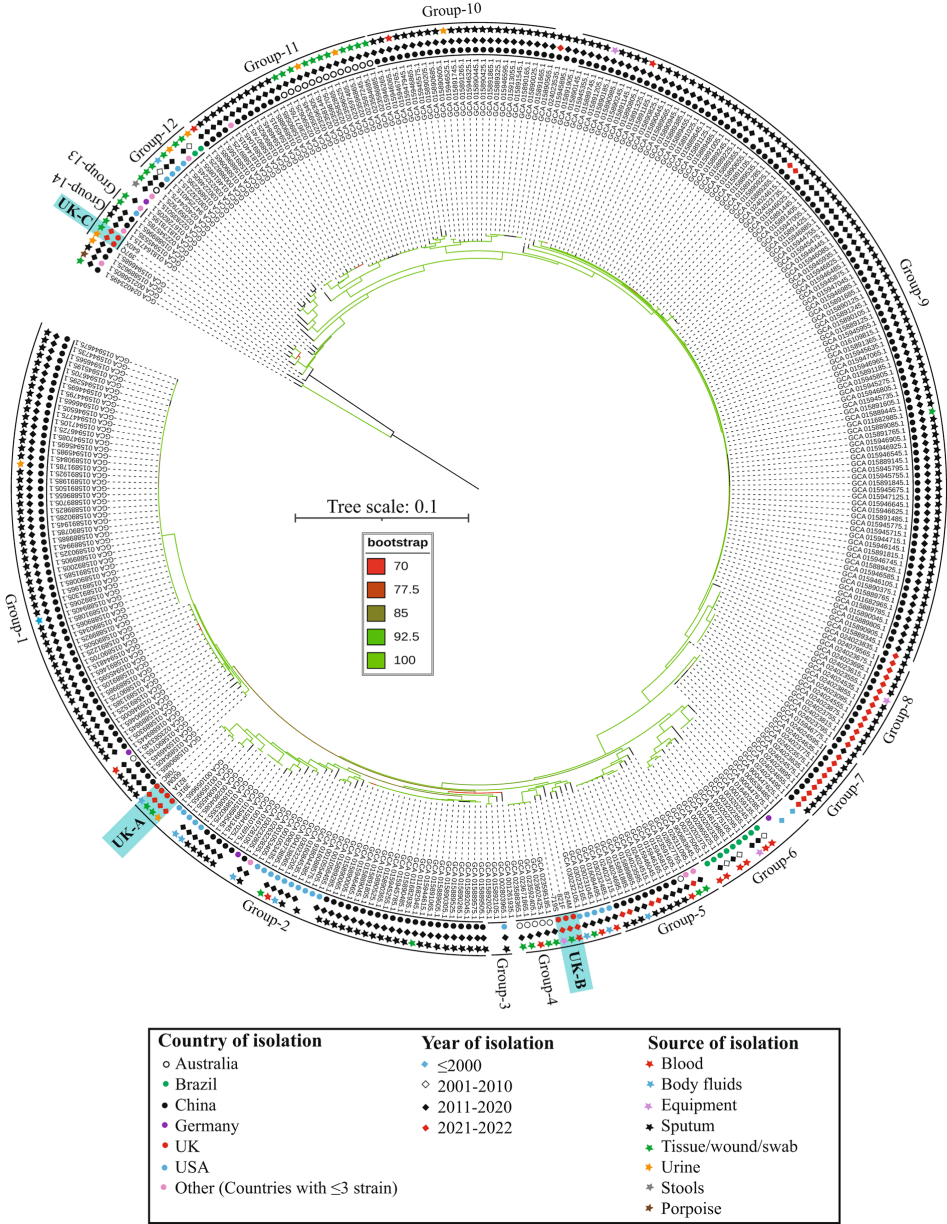
A maximum-likelihood tree from the core genome sequences of *C. striatum* strains. The scale bar represents nucleotide substitutions per site and branch colour reflects bootstrap values. The three subgroups of UK isolates are labelled as UK-A, UK-B and UK-C.

Chinese isolates were highly diverse that predominantly clustered in Group-1, Group-5, Group-7, Group-8, Group-9 and Group-10 and some formed subgroups within Group-2 and Group-11 (Fig. 1), which is consistent with the previous study [13]. Most of Australian strains from infected tissues, wounds or urine samples formed a subgroup within Group-11 (Fig. 1) and blood isolates from Brazil clustered in Group-6. These results potentially indicate geographic clustering among *C. striatum* strains. However, 263 of 353 genomes (74.5%) were submitted from China, mostly isolated from sputum samples (249/263 samples; 94.7%). A global collection with a fair representation of other countries needs to be analysed to confirm this finding.

The UK strains were tested for susceptibility against a set of seven antibiotics by disc diffusion method (Table 2) using the European Committee on Antimicrobial Susceptibility Testing criteria (www.eucast.org/ast_of_bacteria; Accessed on 10/08/2023). All nine strains were sensitive to linezolid and vancomycin but were resistant to clindamycin and penicillin. Only one strain was susceptible to doxycycline, two were susceptible to moxifloxacin and only one strain was resistant to rifampicin (Table 2).

**Table 2:** Antimicrobial resistance genes and resistance profiles of *C. striatum* strains

Strain ID	AMR genes	Resistance
708C	*ampC*, *bla*, *pbp2m, ermX*, *tet(W)*	Clindamycin, Doxycycline, Moxifloxacin, Penicillin
824M	*ampC*, *bla*, *ermX*, *tet(W), aac(3)-XI*	Clindamycin, Doxycycline, Moxifloxacin, Penicillin
600M	*ampC*, *bla*, *pbp2m, ermX*, *tet(W)*	Clindamycin, Doxycycline Moxifloxacin, Penicillin
640X	*ampC*, *bla*, *tet(Z)*	Clindamycin, Doxycycline, Penicillin
719S	*ampC*, *bla*, *pbp2m, ermX*, *tet(W)*	Clindamycin, Doxycycline, Moxifloxacin, Penicillin
821F	*ampC*, *bla*, pbp2m, ermX*, tet(W)	Clindamycin, Doxycycline, Moxifloxacin, Penicillin
821A	*ampC*, *bla*, *pbp2m, ermX, tet(W)*	Clindamycin, Doxycycline, Moxifloxacin, Penicillin, Rifampicin
391E	*ampC*, *bla*, *pbp2m, ermX, tet(W)*	Clindamycin, Doxycycline, Moxifloxacin, Penicillin
397Q	*ampC*, *bla*, *ermX*	Clindamycin, Penicillin

Notes: 1. All strains were tested against Clindamycin, Doxycycline, Linezolid, Moxifloxacin, Penicillin, Rifampicin and Vancomycin.

2. All strains were susceptible to Linezolid and Vancomycin.

3. * = presence of gene confirmed by analysing Illumina reads.

Genome sequences of these strains were analysed using Comprehensive Antibiotic Resistance Database [14] and ResFinder 4.0 [15] with ≥ 80% coverage and identity threshold values to identify antimicrobial resistance genes. Gene *ermX*, associated with resistance to macrolides, lincosamides and streptogramins, and *tet(W)* conferring resistance to tetracyclines [16, 17] were present among six strains (Table 2), explaining the resistance to clindamycin (lincosamide) and doxycycline (tetracycline) [13, 18]. A truncated *ermX* gene was detected in the genome of clindamycin resistant strain 821F, potentially due to ambiguous bases added during scaffolding. The presence of this gene was confirmed by analysing the Illumina reads. However, strain 640X was also found to be resistant to clindamycin despite an absence of *ermX* gene, indicating other potential mechanisms of resistance to lincosamides. ResFinder 4.0 identified an additional gene *aac(3)-XI* in strain 824M, which confers resistance to aminoglycosides [19].

The protein BLAST searches using ≥ 50% query coverage, ≥ 35% sequence identities and an E-value < 10^− 5^ as threshold [20], revealed the presence of a penicillin-binding protein, AmpC (Uniprot accession: A0A076NFW6) and a β-lactamase transpeptidase-like protein, Bla (Uniprot accession: A0A076NFW6) among all nine strains, conferring resistance against β-lactam antibiotics [4, 21]. Six *C. striatum* strains also possessed Pbp2m (GenBank accession: ART21765.1), another penicillin-binding protein associated with penicillin resistance [22]. Mutations in the quinolone resistance-determining region of *gyrA* gene are responsible for resistance to quinolones including moxifloxacin [4]. When compared to GyrA sequence of quinolone-susceptible strain ATCC6940 (GenBank accession: AY559038), seven strains had a serine to valine substitution at position 87 (S87V), with an additional serine to phenylalanine substitution at position 150 (S150F) in four strains. These results indicate the circulation of three distinct MDR lineages within the hospital. However, it is difficult to infer whether these lineages have been recently introduced or are already well-established in the hospital-settings. Therefore, an active surveillance to monitor nosocomial *C. striatum* infections and cross-transmission between patients is needed.

Corynebacterial virulence genes [23–26] were detected using the protein BLAST searches and the virulence factor database [27]. All UK-A and UK-B isolates possessed SpaDEF type-1 and SpaDEF type-2 pilus gene clusters (Table 3). SpaDEF type-1 pilus gene cluster (Fig. 2A) encompassed five genes encoding two sortases (*srtB* and *srtC*), a minor pilin subunit (*spaD*), a major subunit (*spaE*), and a tip protein (*spaF*). However, *spaF* gene in strains 821A and 391E, and *spaE* in 824M were truncated. SpaDEF type-2 cluster only had a single sortase, *srtC* (Fig. 2B). Strain 397Q (UK-C clade) only possessed SpaDEF type-2 cluster whereas SpaDEF type-1 and a novel *spa* gene cluster was observed in strain 640X (Table 3; Fig. 2C). The latter showed similar coverage and identities with genes in SpaGHI and SpaABC type gene clusters and need molecular characterisation. Only SpaDEF type-1 cluster has been previously reported in *C. striatum* [13, 18] and we report additional SpaDEF type-2 and a novel gene cluster among UK isolates. Surface pili facilitate adhesion to the host cells and variation in the number of pilus gene clusters and gain/loss of gene functions will likely contribute to variations in the degree of pathogenesis [28].

**Fig. 2:**
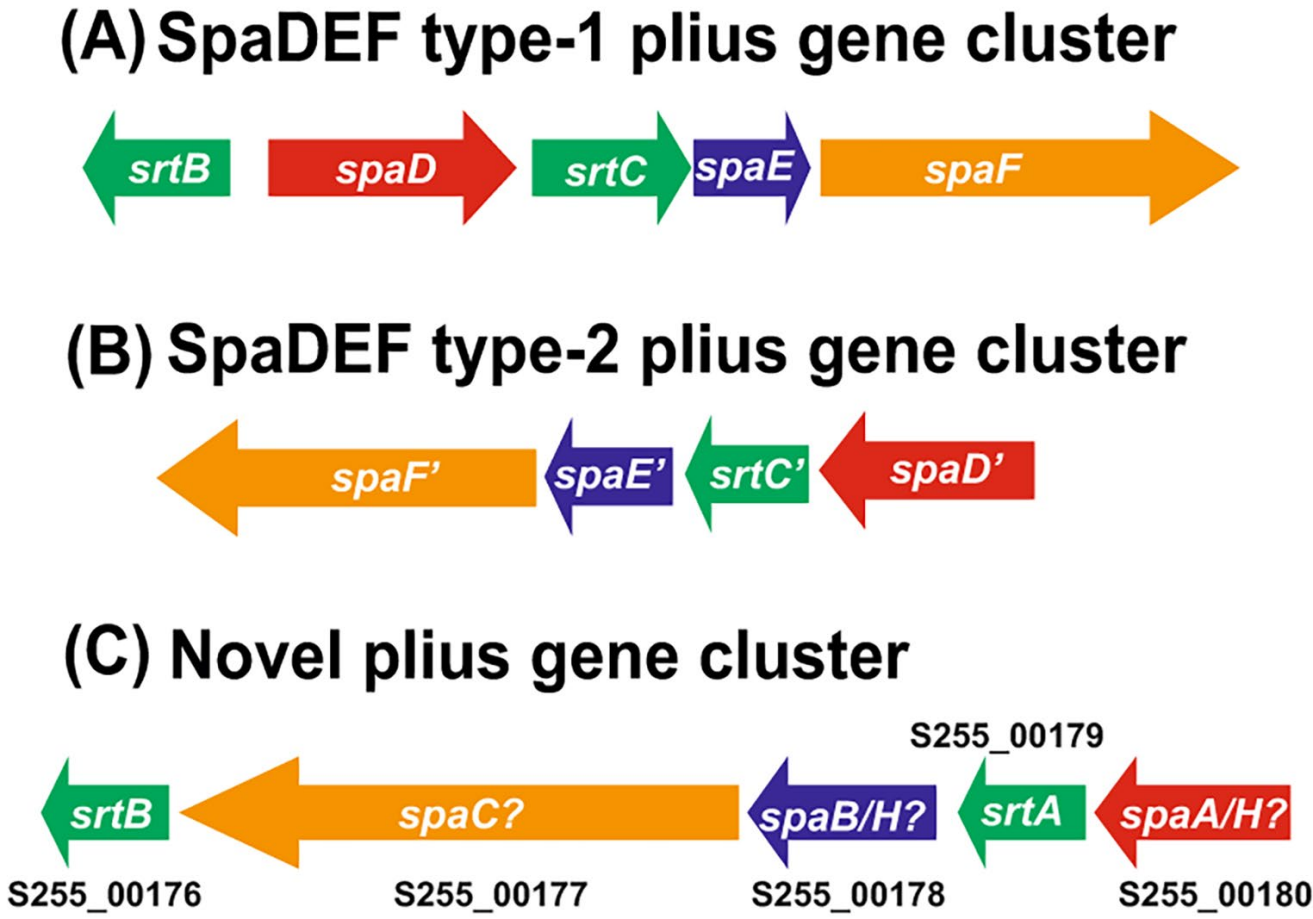
The organisation of pilus gene clusters observed among *C. striatum* strains: **(A)** SpaDEF type-1, **(B)** SpaDEF type-2 and **(C)** a novel gene cluster. Genes encoding minor pilin subunits, major pilin subunits, tip proteins and sortases are shown in red, blue, orange and green colours, respectively. The schematic is not to scale. The locus tags from strain 640X are mapped on the novel gene cluster

**Table 3:** Corynebacterial virulence genes present among *C. striatum* strains

strain	Group (UK lineage)	Spa gene clusters	Corynebacterial virulence genes
708C	Group-2 (UK-A)	SpaDEF type-1, SpaDEF type-2	*accD3, cwlH, dtsR1, dtsR2, mycP, nor, nrpS2, rpfA, rpfB, rpfI, sgnH, vsp2*
600M	Group-2 (UK-A)	SpaDEF type-1, SpaDEF type-2	*accD3, cwlH, dtsR1, dtsR2, mycP, nor, nrpS2, rpfA, rpfB, rpfI, sgnH, vsp2*
821A	Group-2 (UK-A)	SpaDEF type-1^a^, SpaDEF type-2	*accD3, cwlH, dtsR1, dtsR2, mycP, nor, nrpS2, rpfA, rpfB, rpfI, sgnH, vsp2*
391E	Group-2 (UK-A)	SpaDEF type-1^a^, SpaDEF type-2	*accD3, cwlH, dtsR1, dtsR2, mycP, nor, nrpS2, rpfA, rpfB, rpfI, sgnH, vsp2*
824M	Group-4 (UK-B)	SpaDEF type-1^b^, SpaDEF type-2	*accD3, cwlH, dtsR1, dtsR2, mycP, nor, nrpS2, rpfA, rpfB, rpfI, sgnH, vsp2*
719S	Group-4 (UK-B)	SpaDEF type-1, SpaDEF type-2	*accD3, cwlH, dtsR1, dtsR2, mycP, nrpS2, rpfA, rpfB, rpfI, sgnH, vsp2*
821F	Group-4 (UK-B)	SpaDEF type-1, SpaDEF type-2	*accD3, cwlH, dtsR1, dtsR2, mycP, nrpS2, rpfA, rpfB, rpfI, sgnH, vsp2*
397Q	Group-14 (UK-C)	SpaDEF type-2	*accD3, cwlH, dtsR1, dtsR2, mycP, nor, nrpS2, rpfA, rpfB, rpfI, sgnH, vsp2*
640X	Group-14 (UK-C)	SpaDEF type-1, novel Spa type	*accD3, cwlH, dtsR1, dtsR2, mycP, nor, nrpS2, rpfA, rpfB, rpfI, sgnH, vsp2*

^a^ truncated *spaF* gene

^b^ truncated *spaE* gene

Several virulence-associated proteins including acyl-CoA carboxylase β-subunits (DtsR1, DtsR2 and AccD3), cell wall-associated hydrolase (CwlH), nonribosomal peptide synthetase (NrpS2), nitric oxide reductase (Nor), resuscitation-promoting factors RpfA and RpfB, Rpf interacting protein RpfI, subtilisin-like serine protease (MycP), SGNH-hydrolase (SgnH) and a venom serine protease Vsp2 were identified among *C. striatum* strains (Table 3). All these proteins were highly conserved among the UK isolates except for Nor, which was absent in two UK-B strains, 719S and 821F (Table 3). CwlH, RpfI, RpfA and RpfB are important for cell division and cell surface organization and help in adhesion and internalization of the pathogen by the host cells [26, 29–32]. The genes encoding NrpS2 and subunits of acyl/propionyl-CoA carboxylase were upregulated during the macrophage infection [33]. DtsR1, DtsR2, and AccD3 are important for fatty acid and mycolic acid biosynthesis that helps corynebacteria resist the environmental stress [34–36]. Subtilisin proteases are potentially involved response to hypoxic stress during the colonization [37]. Serine protease enzymes are also important for virulence that promote pathogen survival and induction of inflammatory cytokines [38, 39].

Iron uptake genes including *fagABCD*, *hmuTUV*, *irp6ABC* and *irtAB* operons and two exochelin genes [40–42] were variably present among UK isolates (Table S2). Strains in UK-A and UK-B clades possessed two *fagABCD* operons while UK-C strains only possessed one operon. However, additional copies of *fagBD* were observed among all nine strains (Table S2). *irtAB* operon and exochelin genes were also absent among UK-C isolates. This variation potentially suggest that UK-A and UK-B strains are better equipped to scavenge iron from host cells than UK-C strains, as previously reported among *C. diphtheriae* strains [23].

Five transcriptional regulators *dtxR*, *senX3*, *sigA*/*rpoV*, *sigD* and *whiB3* were present among all nine isolates (Table S2). *dtxR* regulates iron metabolism [43], *whiB3* regulates virulence lipid anabolism and modulates macrophage response [44] and *senX3*, *sigA*/*rpoV* and *sigD* are important for stress response and persistence in the host cells [45–47]. Two genes of Pup proteasome system, *pafA* and *mpa*, and *secA2*, a part of the multi-substrate system involved in exporting various substrates [48], were also present among all UK strains (Table S2). Mpa (*Mycobacterium* proteasome ATPase) and PafA (proteasome accessory factor A) provide resistance against reactive nitrogen intermediates produced by macrophages [49, 50]. SecA2 pathway helps with the phagosome maturation arrest [51] and hence, survival and persistence of the pathogen in the host. Therefore, *C. striatum* strains are well equipped to invade and survive the host immune response during infection.

## Conclusions

Three distinct MDR lineages of *C. striatum* are present in a UK hospital. These strains well equipped with virulence genes involved in adhesion, invasion, and survival of the pathogen in the host cells and have potential to establish in the healthcare settings. Therefore, adequate active surveillance strategies need to be developed to monitor and control the potential nosocomial cross-transmission between patients in healthcare settings.

### Electronic supplementary material

Below is the link to the electronic supplementary material.

Supplementary Material 1

Supplementary Material 2

## Data Availability

The final scaffolded genome sequences of *C. striatum* strains have been deposited to the GenBank under the accession numbers JASNMG000000000-JASNMN000000000 and CP142379, and are publicly available.
